# Supplementary material for: COZOID: contact zone identifier for visual analysis of protein-protein interactions
Source: BMC Bioinformatics. 2018 Apr 6;19:125. doi: 10.1186/s12859-018-2113-6 (PMC5889581; doi:10.1186/s12859-018-2113-6)
Supplement: Supplementary file 2 — Software build. Executable binary file of the software tool. (ZIP 118,784 kb) [file 12859_2018_2113_MOESM2_ESM.zip › cozoid/platform/docs/testng-6.5.1-javadoc.zip!/org/testng/IClass.html]

IClass (TestNG)


---


|  |  |  |
| --- | --- | --- |
| **Overview** \| **Package** \| **Class** \| **Tree** \| **Deprecated** \| **Index** \| **Help** | |  |
| **PREV CLASS**   **NEXT CLASS** | **FRAMES**    **NO FRAMES**     **All Classes** |  |
| SUMMARY: NESTED \| FIELD \| CONSTR \| METHOD | DETAIL: FIELD \| CONSTR \| METHOD |  |


---


## org.testng Interface IClass

**All Superinterfaces:**: java.io.Serializable

**All Known Subinterfaces:**: ITestClass

---

``` public interface IClass extends java.io.Serializable ```

`IClass` represents a test class and a collection of its instances.

**Author:**
:   Cedric Beust

---

| **Method Summary** | |
| --- | --- |
| `void` | `addInstance(java.lang.Object instance)` |
| `int` | `getInstanceCount()` |
| `long[]` | `getInstanceHashCodes()` |
| `java.lang.Object[]` | `getInstances(boolean create)` |
| `java.lang.String` | `getName()` |
| `java.lang.Class` | `getRealClass()` |
| `java.lang.String` | `getTestName()`             If this class implements ITest, returns its test name, otherwise returns null. |
| `XmlClass` | `getXmlClass()` |
| `XmlTest` | `getXmlTest()` |

**Method Detail**

### getName

```
java.lang.String getName()
```

:   **Returns:**: this test class name. This is the name of the corresponding Java class.

---


### getXmlTest

```
XmlTest getXmlTest()
```

:   **Returns:**: the <test> tag this class was found in.

---


### getXmlClass

```
XmlClass getXmlClass()
```

:   **Returns:**: the \*lt;class> tag this class was found in.

---


### getTestName

```
java.lang.String getTestName()
```

:   If this class implements ITest, returns its test name, otherwise returns null.

---


### getRealClass

```
java.lang.Class getRealClass()
```

:   **Returns:**: the Java class corresponding to this IClass.

---


### getInstances

```
java.lang.Object[] getInstances(boolean create)
```

---


### getInstanceCount

```
int getInstanceCount()
```

---


### getInstanceHashCodes

```
long[] getInstanceHashCodes()
```

---


### addInstance

```
void addInstance(java.lang.Object instance)
```


---


|  |  |  |
| --- | --- | --- |
| **Overview** \| **Package** \| **Class** \| **Tree** \| **Deprecated** \| **Index** \| **Help** | |  |
| **PREV CLASS**   **NEXT CLASS** | **FRAMES**    **NO FRAMES**     **All Classes** |  |
| SUMMARY: NESTED \| FIELD \| CONSTR \| METHOD | DETAIL: FIELD \| CONSTR \| METHOD |  |


---
